# Is a reproduction number of one a threshold for *Plasmodium falciparum* malaria elimination?

**DOI:** 10.1186/s12936-016-1437-9

**Published:** 2016-07-26

**Authors:** Jamie T. Griffin

**Affiliations:** School of Mathematical Sciences, Queen Mary University of London, Mile End Road, London, E1 4NS UK

**Keywords:** Malaria, Mathematical model, Elimination, Reproduction number

## Abstract

**Background:**

The basic reproduction number (*R*_0_) is an important summary of the dynamics of an infectious disease. It is a threshold parameter: an infection can only invade a population if *R*_0_ is greater than 1. However, a number of studies using simple models have suggested that for malaria, it is in theory possible for infection to persist indefinitely even if an intervention has reduced *R*_0_ below 1. Such behaviour is known as a bistable equilibrium. Using two published mathematical models which have both been fitted to detailed, age-stratified data on multiple outcomes, the article investigates whether these more complex models behave in such a way, and hence whether a bistable equilibrium might be a real feature of *Plasmodium falciparum* malaria in Africa.

**Results:**

With the best-fitting parameter values, neither model has a bistable state, because immunity reduces onwards infectiousness. The results imply that there is a threshold such that if interventions can reduce transmission so that *R*_0_ is below 1 for long enough, then malaria will be locally eliminated.

**Conclusions:**

This means that calculations of the reduction in *R*_0_ that interventions can achieve (the effect size) have a useful and straightforward interpretation, whereas if the theoretical possibility of a bistable equilibrium were the real behaviour, then such effect size calculations would not have a clear interpretation.

**Electronic supplementary material:**

The online version of this article (doi:10.1186/s12936-016-1437-9) contains supplementary material, which is available to authorized users.

## Background

The basic reproduction number *R*_0_ of an infectious disease is defined as the number of secondary cases produced by a typical infected case in an otherwise susceptible population. It is important as a threshold quantity: if *R*_0_ is less than 1 then it is not possible for an infection to invade a susceptible population. The same threshold in many cases also applies in the other direction: starting from an endemic state, if *R*_0_ is reduced below 1 and kept there then the infection will die out. However a number of studies have shown that for malaria, in theory it is possible that there are stable endemic states with *R*_0_ below 1 that can persist indefinitely. This phenomenon is known as a bistable equilibrium since there can be a set of model parameters for which the disease-free equilibrium and an endemic equilibrium are both stable.

Águas et al. [1] formulated a model of malaria transmission with immunity, where the effect of immunity is to reduce the probability of clinical malaria. They used the model to show that if immune individuals have a longer duration of infection, and hence more overall onwards infectiousness than non-immunes, then there are parameter regions with a bistable equilibrium. Keegan and Dushoff [2] further explored the dynamics of a similar model, and derived a quantity based on the ratio of reproduction numbers with and without immunity that is a threshold for whether there is a bistable equilibrium. In a review of countries which had eliminated malaria, Chikaya et al. found that once elimination is reached, a resurgence to high endemic levels is rare, whereas it is common in countries that pushed malaria to low levels without eliminating it [3, 4]. Smith et al. [5] investigated this stability of elimination, looking at the evidence for several possible explanations. One explanation was a bistable equilibrium: it could be that in some endemic settings *R*_0_ was below 1, perhaps having been reduced previously by changes such as improved housing and increased levels of treatment. Then if interventions further reduced transmission for some years, malaria could be eliminated and the population would lose their immunity. When the interventions are removed, the reproduction number will return to the same value it had when there was endemic transmission, but that value is below 1 and so if malaria is imported, it will die out.

In these models of malaria transmission with a bistable equilibrium, partial immunity to malaria has the effect of increasing an individual’s onward transmission over the course of an infection. The most plausible mechanism for this is that immunity reduces the probability of clinical symptoms. With no symptoms and hence no treatment, the infection can persist for longer, whereas effective treatment clears the infection. However, immunity to malaria not only reduces the probability of symptoms, but also lowers parasite densities and hence reduces onwards infectiousness per unit time, and so it is not clear a priori whether overall, immunity increases or reduces onwards transmission. This article investigates whether models which capture immunity in a more realistic way show bistability, using two mathematical models which both incorporate immunity at several stages, and which have both been fitted to a wide range of types of data on *Plasmodium falciparum* malaria in Africa, but which have quite different internal structures.

## Methods

### Transmission models

Two models of human infection and immunity are considered. The first is the model of Griffin et al. [6, 7], which is referred to here as the Griffin model. It is implemented as both a deterministic compartmental model that was fitted to data and a corresponding individual-based model for flexibility in looking at the impact of interventions: the former is used for the results here. The second model is the OpenMalaria model described in [8–11] and summarized with updated fitted parameter values in [12].

For the results in this paper, for simplicity each human model is combined with the same standard model of the mosquito stages of the transmission cycle, with a single mosquito species. The fitting to data of the OpenMalaria model was of the human model with a forced input entomological inoculation rate (EIR, the rate at which people are bitten by infectious mosquitoes), and so it can be combined with any vector model. There is a mosquito density per person *m*, death rate *μ* and a fixed delay from infection to becoming infectious of length *τ*. The dynamics of mosquito infection at time *t* are given by

1 $$\begin{aligned} \frac{{dS_{M} }}{dt} &= \mu m - \varLambda_{M} \,S_{M} - \mu S_{M} \hfill \\ \frac{{dE_{M} }}{dt} &= \varLambda_{M} \,S_{M} - \varLambda_{M} (t - \tau )S_{M} (t - \tau )e^{ - \mu \tau } - \mu E_{M} \hfill \\ \frac{{dI_{M} }}{dt} &= \varLambda_{M} \,(t - \tau )\,\,S_{M} (t - \tau )e^{ - \mu \tau } - \mu I_{M} \hfill \\ \end{aligned}$$

Λ_*M*_ is the force of infection experienced by mosquitoes, and *S*_*M*_, *E*_*M*_ and *I*_*M*_ are the numbers (per person) which are susceptible, latently infected and infectious, respectively.

### Reproduction number formulae

If there is no variation in the biting rate or any other factors between people, then the basic reproduction number is given by

$$R_{0} = \frac{{m\alpha^{2} e^{ - \mu \tau } bC}}{\mu } = m\gamma \alpha^{2} bC$$

*α* is the biting rate on humans and *γ* = e^−*μτ*^⁄*μ* is the expected duration of infectiousness of an infected mosquito. *b* is the probability of infection if bitten by an infectious mosquito. *C* is the expected overall human infectiousness to mosquitoes if infected, integrating over the human infectious period. For example if there is a human infectious period of average length *d* with a constant probability *c* of infecting each susceptible mosquito that bites during this time, then *C* = *dc*. The vectorial capacity is *V* = *mγα*^2^, and *R*_0_ = *VbC*.

To extend this to include variation between people, suppose that the human population is divided into *n* groups, with a proportion *p*_*i*_ in group *i* who have biting rate *α*_*i*_, probability of infection *b*_*i*_ and expected overall onward infectiousness *C*_*i*_. This variation could include variation by age, in which case people are grouped according to their age at infection. Continuous variation in one or more dimensions can be approximated arbitrarily closely in this way. The expected number of humans in group *i* that an infected mosquito will infect is *γp*_*i*_*α*_*i*_*b*_*i*_, and the expected number of mosquitoes infected by an infected human in group *i* is *mα*_*i*_*C*_*i*_. So *R*_0_, which is the expected number of mosquitoes infected by an infected mosquito via a single human infection, can be calculated by summing over the possible types of intermediate human:

2 $$\begin{aligned} R_{0} &= \sum\limits_{i = 1}^{n} {\gamma p_{i} \alpha_{i} b_{i} \;m\alpha_{i} C_{i} } \\ &= m\gamma \sum\limits_{i = 1}^{n} {p_{i} \alpha_{i}^{2} b_{i} } C_{i} = m\gamma E_{H} \left( {\alpha^{2} bC} \right)\end{aligned}$$

where *E*_*H*_() denotes the expected value over humans. The same result can be derived using the next generation method [13] by forming a matrix with mosquitoes and the *n* types of humans as separate generations, finding the largest eigenvalue *λ*, and taking *R*_0_ = *λ*^2^. For the Griffin model, *E*_*H*_(*α*^2^*bC*) with no immunity can be found using explicit formulae, whereas for the OpenMalaria model the individual-based simulation was used. Details for both models are given below.

For all the results in this paper, the reproduction number may include treatment for symptomatic malaria, which changes the onward infectiousness *C* by clearing some infections. As treatment could be considered as an intervention, the term *R*_C_ for “reproduction number under control” could be used in these cases, but for simplicity the term *R*_0_ has been used throughout. It is necessary to include treatment in the model as it is treatment which in some models may lead to bistable behaviour.

### Deriving R_0_ from the endemic equilibrium

It is assumed that the quantity which varies to give different values for *R*_0_ and the EIR is the mosquito density per person *m*. For a given EIR *ε*, the human equilibrium states can be found. For the Griffin model, which is a compartmental model stratified by age and biting rates, this can be done analytically, as described in Additional file 1. For the individual-based OpenMalaria model it is done by running the simulation model with a forced, fixed EIR over one human lifetime with a large enough population size that stochastic variation is negligible.

At this equilibrium, the force of infection on mosquitoes Λ_*M*_ can be calculated or output from the simulation model. Then the proportion of mosquitoes which are infectious follows from the equilibrium solution of the mosquito model of Eq. (1).

$$\frac{{I_{M} }}{m} = \frac{{\Lambda_{M} e^{ - \mu \tau } }}{{\Lambda_{M} + \mu }}$$

Substituting *I*_*M*_ back into a formula for the EIR gives an expression for *m* and hence *R*_0_

$$\begin{aligned} \varepsilon &= I_{M} \alpha_{0} = m \alpha_{0} \frac{{\Uplambda_{M} e^{ - \mu \tau } }}{{\Uplambda_{M} + \mu }} \\ m &= \frac{{\varepsilon (\Uplambda_{M} + \mu )}}{{\alpha_{0} \Uplambda_{M} e^{ - \mu \tau } }} \\ m\gamma &= \frac{\varepsilon }{{\alpha_{0} \Uplambda_{M} }}\left( {\frac{{\Uplambda_{M} }}{\mu } + 1} \right) \\ \end{aligned}$$

3 $$\begin{aligned} R_{0} &= m\gamma E_{H} \left( {\alpha^{2} bC} \right) \\ &= \frac{\varepsilon }{{\alpha_{0} \Uplambda_{M} }}\left( {\frac{{\Uplambda_{M} }}{\mu } + 1} \right)E_{H} \left( {\alpha^{2} bC} \right) \\ \end{aligned}$$

Note that Λ_*M*_ is found from the endemic equilibrium, but *E*_*H*_(*α*^2^*bC*) is for people with no immunity. *ε* here is the mean EIR experienced by the population, not the EIR for adults.

### Details of R_0_ for the Griffin model

In the Griffin model, each individual has a relative biting rate *ζ* which has a mean of 1 and probability distribution *h*(*ζ*), and is assumed to be fixed over time. The biting rate at age *a* is *ζψ*(*a*)*α*_0_/*ω*, where *α*_0_ is the mean biting rate. *ψ*(*a*) approaches 1 at older ages, and *ω* is a normalising constant for the biting rate by age. Let *g*(*a*) be the probability distribution of ages in the population. In this model, with no immunity, *b* and *C* do not vary by age and so

4 $$\begin{aligned} R_{0} &= m\gamma E_{H} \left( {\alpha^{2} bC} \right) \\ &= m\gamma bC\int\limits_{0}^{\infty } {\int\limits_{0}^{\infty } {\left( {\zeta \psi (a)\alpha_{0} /\omega } \right)^{2} h(\zeta )g(a)\;d\zeta \;da} } \\ &= m\gamma \alpha_{0}^{2} bC\delta_{a} \delta_{h} \\ \end{aligned}$$

$$\begin{aligned} \delta_{a}& = \int\limits_{0}^{\infty } {\left( {\psi (a)/\omega } \right)^{2} g(a)\;da} ,\;\delta_{h} = \int\limits_{0}^{\infty } {\zeta^{2} h(\zeta )\;d\zeta } \\ \omega &= \int\limits_{0}^{\infty } {g(a)\psi (a)\;da} \\ \end{aligned}$$

*δ*_*a*_ and *δ*_*h*_ are corrections due to the variation in biting rates by age and other heterogeneity which both increase *R*_0_.

With an exponential age distribution with mean 1/*η*, so that *g*(*a*) = *η*e^−*ηa*^ and taking $\psi (a) = \left( {1 - \rho \text{e}^{{ - a/a_{0} }} } \right)$,

$$\begin{aligned} \omega &= \int\limits_{0}^{\infty } {g(a)\psi (a)\;da} = 1 - \frac{\rho \eta }{{\eta + 1/a_{0} }} \\ \;\delta_{a} &= \int\limits_{0}^{\infty } {\left( {\psi (a)/\omega } \right)^{2} g(a)\;da} = \frac{1}{{\omega^{2} }}\left( {1 - \frac{2\rho }{{1 + 1/(\eta a_{0} )}} + \frac{{\rho^{2} }}{{1 + 2/(\eta a_{0} )}}} \right) \\ \end{aligned}$$

Taking 1/*η* as 21 years, to approximate the cross-sectional age distribution in sub-Saharan Africa, with *ρ* = 0.85 and *a*_0_ = 8 years, gives *δ*_*a*_ = 1.1.

Griffin et al. [6] assume that biting rates vary between people according to a log-normal distribution with a mean of 1 and variance on the log scale of *σ*^2^ = 1.67. This gives

$$\delta_{h} = \int\limits_{0}^{\infty } {\zeta^{2} h(\zeta )\;d\zeta } = e^{{\sigma^{2} }} = 5.31$$

So this amount of heterogeneity in biting increases *R*_0_ by more than fivefold for a given value of *m*.

**Fig. 1:**
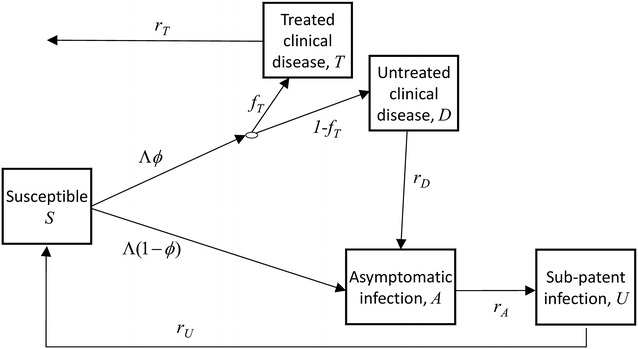
Progression of a single blood stage infection with force of infection Λ in the model of Griffin et al. [6]

The model structure is shown in Fig. 1. The force of infection (the rate at which new infections occur) is Λ. A new infection leads to disease with probability *ϕ* and there is effective treatment of symptomatic malaria with probability *f*_*T*_. *C* can be found by adding up the probability of entering each state following infection, multiplied by the expected time spent in that state and by the infectiousness while in the state:

5 $$\begin{aligned} C &= \phi f_{T} \frac{{c_{T} }}{{r_{T} + \eta }} + \phi (1 - f_{T} )\frac{{c_{D} }}{{r_{D} + \eta }} \\ & \quad + \left( {1 - \phi + \phi (1 - f_{T} )\frac{{r_{D} }}{{r_{D} + \eta }}} \right)\left( {\frac{{c_{A} }}{{r_{A} + \eta }} + \frac{{r_{A} }}{{r_{A} + \eta }}\frac{{c_{U} }}{{r_{U} + \eta }}} \right)\end{aligned}$$

Each *r* is the recovery rate from the corresponding model state, and each *c* is the infectiousness of a state, with subscripts *T*, *D*, *A* and *U* referring to the states shown in Fig. 1. With no immunity, *c*_*A*_ = *c*_*D*_.

Equations (4) and (5) ignore the fact that during an infection, individuals age and so the rate at which they are bitten by mosquitoes changes. Correctly accounting for ageing during an infection changes *R*_0_ by around 1 %, but greatly complicates the formulae when there are multiple infection states since it removes the separation between time since infection and age. The formulae that correctly account for ageing are given in Additional file 1, and were used for the results.

The force of infection on mosquitoes at the endemic equilibrium is found by calculating the equilibrium proportions in each state, stratified by age and exposure to mosquitoes, and summing their infectiousness to mosquitoes, weighted by the relative biting rates.

### Checking stability in the Griffin model

Once the equilibrium states were found in the Griffin model for a given EIR *ε*_0_, the equilibrium was checked for stability by randomly perturbing the model states by a small amount and then numerically solving the model differential equations to see whether the system returned to the staring equilibrium or to a different equilibrium. At equilibrium, all the human and mosquito model states were multiplied by 1 + *z*, where *z* is a random draw from a normal distribution with mean zero and standard deviation 0.01 (different for each age group, heterogeneity level and state). The human model states were renormalized to have the correct age distribution. The differential equations were then run for 2000 years, and the EIR implied by the final model states was calculated, denoted by *ε*_1_: such a long time is needed because near the boundary between stable and unstable regions, the model may be extremely slow to either move away from or return to the original equilibrium. For each parameter set and starting EIR this was repeated five times. If the maximum value over the five trials of |log(*ε*_1_/*ε*_0_)| was less than 0.003, then the equilibrium was considered stable, otherwise it was unstable. This criterion was chosen by trial and error as it discriminates well between stable and unstable regions.

### Details of R_0_ for the OpenMalaria model

In the OpenMalaria model, as well as variation in the biting rate by age and in some model variants between people, *C* also varies by age: there is maternal immunity which reduces the parasite density and which is taken to be independent of transmission intensity, and so for the model as implemented this is present even in populations with no prior exposure; the incidence of severe malaria and its case fatality also vary by age, even with no immunity, and the resulting mortality reduces the expected infectiousness; and for *R*_0_ with treatment, there is additional variation in *C* because the probability of clinical malaria and hence treatment varies by age due to an age-dependent parasite density threshold determining the probability of symptoms (the pyrogenic threshold).

With no immunity, all infectious bites result in infection in this model when only considering a single infectious bite per person, which is the case when calculating *R*_0_, and so *b* = 1. (The probability of infection per infectious bite is assumed to decrease if people receive many infectious bites per unit time). *C* was found for a range of age groups by running the simulation model with no transmission, infecting all individuals at time 0, and following them over time to calculate their infectivity to mosquitoes at each time-step (but still not allowing any onward transmission), storing the output by date of birth. Variation in the biting rate by age and due to other factors were included by outputting these quantities for each individual in the population, and then these were combined to find *E*_*H*_(*α*^2^*bC*).

To find the force of infection on mosquitoes at the endemic equilibrium, as one of its standard outputs the OpenMalaria simulation model calculates the mean infectiousness of the population to mosquitoes weighted by availability to mosquitoes, denoted by *κ*. From this, Λ_*M*_ follows at once

$$\Lambda_{M} = \alpha_{0} \kappa$$

## Results

**Fig. 2:**
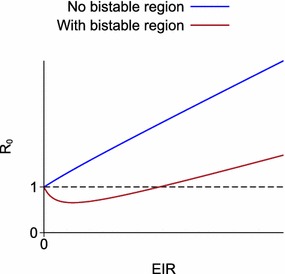
Qualitative behaviour of the theoretical models illustrating bistable behaviour [1, 2]. *R*_0_ plotted against equilibrium EIR

The models of Águas et al. and Keegan and Dushoff show two types of behaviour depending on the parameter values [1, 2]. Figure 2 illustrates these, plotting *R*_0_ against the EIR. If people with immunity are more infectious than those without immunity by a sufficiently large margin, then *R*_0_ initially dips below the line *R*_0_ = 1 as the EIR increases from 0 before later increasing, and so there is a bistable region. Otherwise, *R*_0_ increases monotonically from EIR = 0, remaining above 1 for all positive values of the EIR, and there is no bistable region. For both of these models, there are simple formulae that determine which type of behaviour occurs.

For more complex models with multiple types of immunity, there is no simple formula that will tell us whether there is a bistable region. However it is still possible to see how a model behaves by plotting *R*_0_ against the EIR as in Fig. 2. Two models of *Plasmodium falciparum* malaria are considered which include several kinds of immunity and which have both been fitted to detailed, age-stratified data on multiple epidemiological outcomes. The first is the model described of Griffin et al. [6]. This model is referred to as the Griffin model. The other model is the individual-based OpenMalaria model developed at the Swiss Tropical and Public Health Institute [8–12], whose source code has been made available for download [14].

For both of these models, there is a unique equilibrium state corresponding to any given EIR. It is assumed that the quantity which varies to give different values for the EIR and *R*_0_ is the mosquito density per person *m*. There is always a single value of *m* and *R*_0_ for any given EIR, but there may be more than one EIR that implies the same value of *m* and *R*_0_ (while keeping the model parameters other than *m* fixed). If *R*_0_ is a non-monotonic function of the EIR then there is a bistable region; whereas if *R*_0_ is an increasing function of the EIR and is above 1 for any positive EIR then there is no bistable region.

The results here use the same mosquito model combined with both human models, and so the calculated values of *R*_0_ for the OpenMalaria model will differ a little from those implied by the mosquito model developed by Chitnis et al. [15]. However this will not affect the qualitative pattern of how *R*_0_ varies with the EIR, which is determined by human immunity. All results are for non-seasonal settings, and *R*_0_ is calculated with the same probability of treatment for clinical malaria as is used to find the endemic equilibrium states.

### Griffin model

**Fig. 3:**
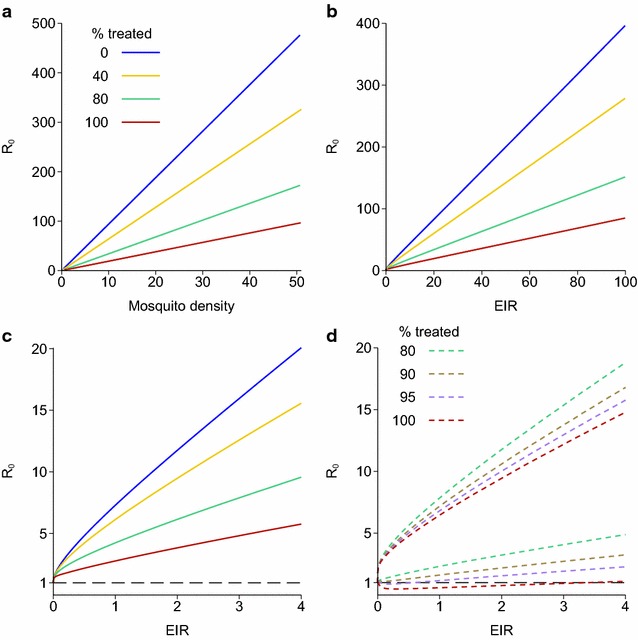
Reproduction number, EIR and mosquito density for the endemic equilibrium of the model of Griffin et al. [6]. Each *line* shows a different proportion of clinical cases being treated. **a** *R*_0_ plotted against mosquito density per person *m*; **b** *R*_0_ plotted against EIR; **c** *R*0 plotted against EIR—the same data as **b**, but only showing low EIRs; **d**: 95 % credible intervals for *R*0 based on the posterior distribution of the fitted human model parameters, plotted against EIR for high treatment levels

The Griffin model was fitted to data using Bayesian methods, resulting in a posterior distribution that accounts for the uncertainty in the human model parameters. The model outputs shown are the posterior median over 30,000 parameter sets from this distribution, with 95 % credible intervals on some plots. For a given mosquito density, *R*_0_ is greatly reduced by high treatment levels (Fig. 3a). With no treatment, *R*_0_ is roughly four times the EIR at medium to high transmission. The posterior median *R*_0_ remains above 1 for any positive EIR, at a range of treatment levels from none to 100 % of symptomatic malaria cases being effectively treated (Fig. 3b, c). So the best estimate from this model is that there is no bistable region for any level of treatment. At very high treatment levels, the lower 95 % credible interval for *R*_0_ does go below 1, but this only happens if at least 95 % of symptomatic cases are effectively treated (Fig. 3d).

**Fig. 4:**
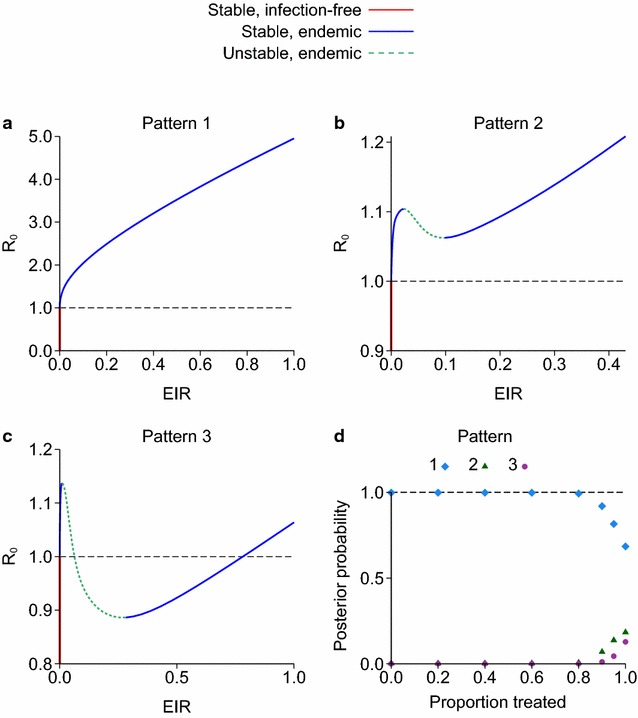
**a**–**c** Three patterns of how *R*_0_ changes as a function of the equilibrium EIR for the model of Griffin et al. [6], showing which equilibria are stable or unstable. **d** Posterior probability of each pattern plotted against the proportion of clinical cases treated

The model behaviour for each parameter set from the posterior distribution can be classified according to how *R*_0_ varies as a function of the EIR. These fell into one of three patterns, illustrated in Fig. 4a–c:

1. *R*_0_ monotonically increases with EIR;
2. *R*_0_ increases, decreases and then increases, but is always above 1 for any positive EIR;
3. *R*_0_ increases, decreases and then increases, and goes below 1 for some positive EIRs.

A small number (fewer than 0.1 %) of parameter sets had additional small wiggles in these curves. The posterior probability of each pattern can be calculated by counting how many parameter sets show each type of behaviour. If the proportion of clinical cases treated is up to 80 %, then the posterior probability that *R*_0_ monotonically increases with EIR is over 99 % (Fig. 4d). The posterior probability of pattern 3, where *R*_0_ is below 1 for some EIRs, is 1, 4 or 13 % if 90, 95 or 100 % of cases are effectively treated, respectively. The stability of each equilibrium state was tested as described in the “Methods” section. For all three patterns, regions where *R*_0_ was increasing as a function of EIR were stable and those where *R*_0_ was decreasing were unstable. Both patterns 2 and 3 have a bistable region, i.e. a range of values of *R*_0_ with two stable states. For *R*_0_ > 1, the bistable region consists of two stable endemic states, whereas for *R*_0_ < 1 it consists of a stable endemic state plus the infection-free equilibrium.

For all parameter sets and all treatment levels, in this model *R*_0_ increases above 1 for small EIRs, i.e. there is a forward bifurcation, even for parameter sets that do have a bistable region. So a local stability analysis at small EIRs around the region *R*_0_ = 1 would not have revealed any bistable behaviour. This is in contrast to the models illustrated in Fig. 2, in which *R*_0_ either increases monotonically (a forward bifurcation with no bistable region) or initially decreases below 1 at low EIRs before then increasing (a backward bifurcation and with a bistable region), and so a stability analysis at small EIRs would detect the bistability.

### OpenMalaria model

**Fig. 5:**
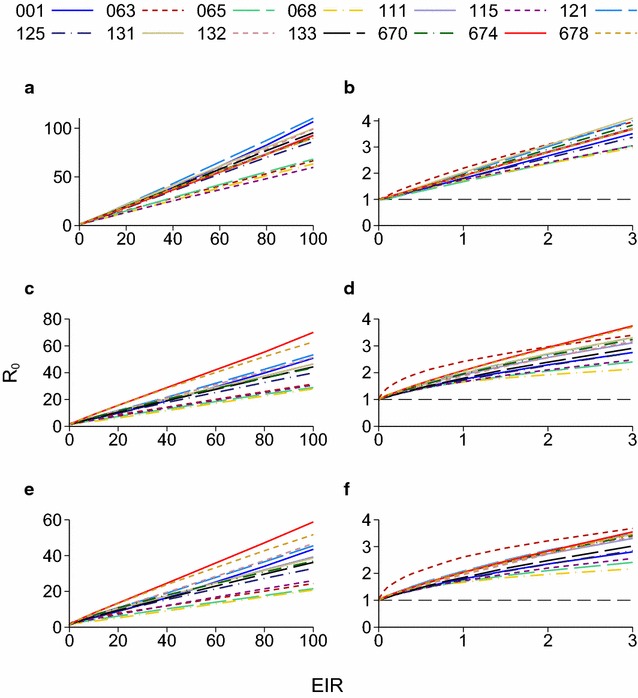
Reproduction number plotted against EIR for the endemic equilibrium of the OpenMalaria model. In the figure legend, the model numbers are as in Table 2 of [12]: 14 different model variants were fitted to data. **a**, **b** No treatment; **c**, **d** 40 % treatment probability in each 5 day period in which there is clinical malaria; **e**, **f** 100 % treatment probability. **b**, **d** and **f** are the same as **a**, **c** and **e** but only show low EIRs

For the OpenMalaria model, 14 model variants have been fitted to data with different assumptions about factors such as heterogeneity in exposure to mosquitoes and the duration of immunity, detailed in [12]. The model is implemented as an individual-based simulation with a 5 day time-step. Treatment of symptomatic malaria is modelled as occurring with a given probability in each time-step, so the treatment level does not have the same interpretation as in the Griffin model. Stochastic variation made estimates of *R*_0_ imprecise for annual EIRs below 0.05. For all the OpenMalaria model variants and all treatment levels, *R*_0_ remains above 1 and is an increasing function of the EIR for any EIR above 0.05, implying that there is no bistable region (Fig. 5). There are no posterior distributions of parameter values available to assess the uncertainty in each model variant.

### Infectious reservoir

**Fig. 6:**
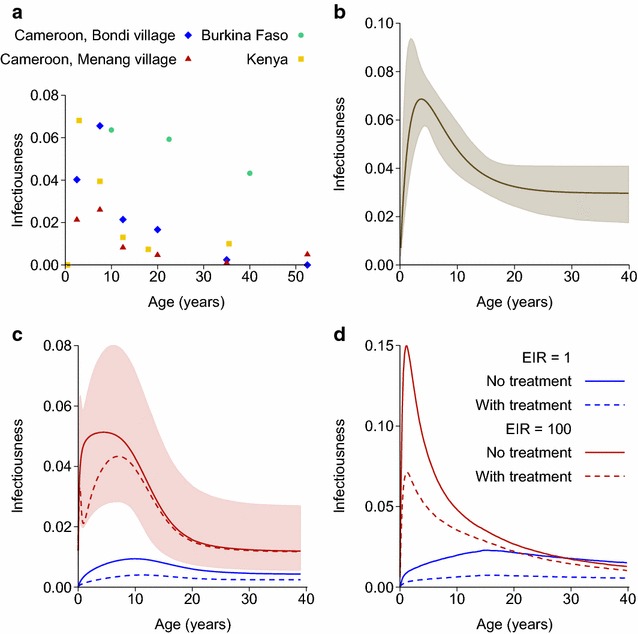
Probability of infecting a susceptible mosquito if bitten, plotted against age. **a** Data from mosquito feeding studies in areas of high transmission. Burkina Faso—annual EIR between 25 and 350 [16]; Cameroon—EIR of around 170 in both villages [17]; Kenya—parasite prevalence above 80 % in children [18]. **b** Estimated using an empirical model fitted to feeding studies and data on gametocytes and asexual parasites [19]. **c** Griffin model at EIRs of 1 or 100, with no treatment or 80 % treatment probability per symptomatic infection. The shaded area is the 95 % credible interval for an EIR of 100 and 80 % treatment. **d** OpenMalaria base model (model 001) at EIRs of 1 or 100, with no treatment or 40 % treatment probability per 5 day time-step

The pattern of infectiousness to mosquitoes with age in endemic areas is also informative about how immunity affects onwards transmission. In several studies in areas of high transmission, people were selected regardless of their infection status and mosquitoes were fed on them or their blood to see what proportion of mosquitoes became infected [16–18]. The result is generally that there is a sharp drop in infectiousness during adolescence (Fig. 6a), meaning that young children are the most infectious, averaging over those who are and are not currently infected, despite also being the most susceptible to clinical malaria and hence likely to have their infections cleared by treatment. The study by Boudin et al. [16] shows a smaller drop in infectiousness with age: this study from 1993 used membrane feeding whereas the others used direct feeding on participants, and it is possible that membrane feeding techniques at that time were not completely standardised. Churcher et al. [19] found a decrease with age by fitting an empirical model to mosquito feeding studies combined with data on gametocytes and asexual parasites from a high transmission area of Burkina Faso (Fig. 6b). Gametocytes are the sexual stages of the malaria parasite that appear in the blood after the asexual stages.

Both the Griffin and OpenMalaria transmission models also show a decrease in infectiousness with age (Fig. 6c, d). Only the base OpenMalaria model is plotted in this figure, but the other model variants show the same pattern. The Griffin model has an empirical function to capture immunity that reduces the detectability of infections, and hence reduces the recorded parasite prevalence. Infectiousness is modelled as a transformation of the probability of detection, with the model fitted to the feeding studies plotted in Fig. 6a as well as to data on parasite prevalence by age from a much wider range of settings. Infectiousness in the OpenMalaria model is modelled differently, and is based on different data: this model explicitly tracks parasite densities as they change during an infection, along with the reduction in these densities due to immunity. Infectiousness to mosquitoes as a function of asexual parasite densities was fitted to data from malaria-therapy studies, in which non-immune patients were infected with malaria as a treatment for syphilis, and also took part in mosquito feeding studies [10].

## Discussion

Previous work has shown that for malaria, in theory it is possible for *R*_0_ to be reduced below 1 but for infection to persist indefinitely, if there is a bistable equilibrium. The results presented here suggest that *P. falciparum* does not behave in this way, and the most likely behaviour is that *R*_0_ increases monotonically as the EIR increases, and is above 1 for all positive EIRs. This implies that the value *R*_0_ = 1 is a threshold for malaria elimination, i.e. starting from an endemic state, if interventions can reduce *R*_0_ below 1 and keep it there for long enough, then the infection will be locally eliminated unless there is re-importation.

The uncertainty in these conclusions can be assessed for the Griffin model using the posterior distribution of human model parameters. For treatment levels above 80 %, the results become less certain, but there is only a substantial posterior probability (above 5 %) of the existence of endemic states with *R*_0_ below 1 if more than 95 % of clinical cases are effectively treated, and even with 100 % treatment a monotonically increasing *R*_0_ is more likely. For the OpenMalaria model the uncertainty was assessed in a different way, as there are 14 different model variants. All of these behaved in the same way, with *R*_0_ an increasing function of the EIR for all treatment levels, and so there were no endemic states with *R*_0_ below 1.

The results in this paper include the same probability of treatment for symptomatic malaria in the calculation of *R*_0_ as is used to calculate the endemic equilibrium states. The reason for this is that treatment for symptomatic malaria is conceptually different to other interventions: one would try to treat people’s symptoms regardless of the transmission setting, but would take the setting into account when considering other interventions. For example when moving towards elimination, if malaria has been locally eliminated in a certain area, policy makers may consider scaling back some interventions and switching to a more reactive policy. But they would not scale back treatment for symptomatic malaria, and so the reproduction number with treatment is the relevant metric.

The methods used here rely on finding the model equilibrium state. Hence the results are a simplification as they do not account for the dynamic changes over time in immunity that would occur if transmission was rapidly reduced [20]. Depending on how immunity alters someone’s onward infectiousness, it is possible that there would be a period after transmission has been reduced when the population still has immunity from the pre-intervention period and that elimination would be possible during this time, even if the reproduction number with the intervention is greater than 1. As the results are for equilibrium states, they also do not include seasonality in transmission.

Of the previously published models which suggested there could be a bistable equilibrium, the model of Águas et al. was fitted to data on the age patterns of severe malaria, while the models of Keegan and Dushoff and Smith et al. were not fitted to data but explored the model behaviour over a range of parameter values [1, 2, 5]. None of the models were fitted to data on infectiousness to mosquitoes, or used any data on parasite prevalence or parasite densities as these vary by age and transmission intensity. All three models showed regions of parameter space with and other regions without a bistable equilibrium. These models all have a single type of immunity. This is modelled in different ways but in all cases, the average duration of infection with immunity is increased compared to non-immunes. Keegan and Dushoff also allow the relative infectiousness of immunes and non-immunes to vary when exploring the model behaviour. They derive a threshold that determines whether or not there is a bistable equilibrium: this occurs if immunes are more infectious over the course of their infection than non-immunes by a sufficiently large margin. The model of Águas et al. behaves in a similar way. It is difficult to determine directly from currently available data whether the assumptions that lead to bistable behaviour are satisfied.

The results in the present paper are based on two models which include immunity at multiple points, and have been fitted to a wide range of different types of data. In particular, as well as having immunity which reduces the probability of symptoms, both models allow for a lower infectiousness per unit time in infected people with some immunity compared to infected people with no immunity. The model structures for onward infectiousness to mosquitoes and how this varies with past exposure and for immunity to clinical malaria differ between the two models: these are the main aspects that determine whether there is a bistable region. The OpenMalaria model explicitly tracks individuals’ parasite densities, which are decreased by immunity. Symptomatic malaria occurs with a certain probability that depends on the parasite density, and the probability of infecting a mosquito also depends on the recent parasite density, and so both of these probabilities are reduced because immunity reduces the parasite density. The Griffin model instead uses separate empirical functions for each kind of immunity. The data the models were fitted to are also different, in particular for onward infectiousness to mosquitoes. These differences make the findings more robust than if the results were only supported by a single model.

For understanding whether there is a bistable equilibrium, some information about immunity when there is low transmission intensity is needed. The datasets that the two models were fitted to were from a range of transmission intensities, although there were more sites with moderate to high transmission. The Griffin model was fitted to data on the age patterns of clinical malaria and of parasite prevalence in sites with parasite prevalence in 2–10 years olds ranging from below 1 to 90 %. Furthermore, people acquire immunity over time as their cumulative exposure increases, and so since there was age-stratified data on multiple end-points, these age patterns carry some additional information about how immunity changes with exposure. Most importantly, older children and adults are less infectious to mosquitoes than young children, averaging over those with detectable infections and those without. This suggests that in terms of the overall contribution to onwards infectiousness, the effect of immunity in reducing parasite densities and hence reducing onward infectiousness when infected, outweighs the effect of a reduction in the probability of symptoms and hence of the infection being cleared by treatment.

## Conclusions

These results are important for using mathematical models to assess the contribution that interventions could make to elimination. The relative change in *R*_0_ due to interventions is known as the effect size [21, 22], and is often straightforward to calculate. The theoretical studies which suggested there was bistable behaviour and stable equilibrium states with *R*_0_ below 1 cast doubt on the usefulness of such effect size calculations because if that prediction was correct then *R*_0_ = 1 would not be a threshold for elimination.

## Additional files

10.1186/s12936-016-1437-9 Details of the methodology.
10.1186/s12936-016-1437-9 R code [23] that can be used to calculate the equilibrium for the model of Griffin et al. plus a sample from the posterior distribution of model parameters.
