# Supplementary material for: Is a reproduction number of one a threshold for Plasmodium falciparum malaria elimination?
Source: Malar J. 2016 Jul 26;15:389. doi: 10.1186/s12936-016-1437-9 (PMC4960803; doi:10.1186/s12936-016-1437-9)
Supplement: Supplementary file 1 — 10.1186/s12936-016-1437-9 Details of the methodology. [file 12936_2016_1437_MOESM1_ESM.pdf]

Is a Reproduction Number of One a Threshold for Plasmodium Falciparum Malaria Elimination?

Jamie T Griffin

School of Mathematical Sciences, Queen Mary University of London, Mile End Road, London, E1 4NS

j.griffin@qmul.ac.uk

Supplementary methods

Equilibrium for the Griffin model

Heterogeneity in exposure

In the model of Griffin et al., the EIR varies between people according to a random variable ζ which does not change during their lifetime: if the mean EIR for adults in the population is ε_0, then the EIR for an adult with heterogeneity level ζ is $\zeta\varepsilon_0$ [1]. ζ has probability density $h(\zeta)$ with a mean of 1, taken to be a log-normal distribution. To model this heterogeneity distribution using a compartmental model, model states are stratified into n exposure categories. Let $x_1,\ldots,x_n$ and $w_1,\ldots,w_n$ be the Gauss-Hermite integration points and weights for integrating a function multiplied by a standard normal probability density [2]. A proportion w_j of the population are in exposure category j and the relative biting rate in this category is

$$\zeta_j = \exp\left(-\sigma^2/2 + \sigma x_j\right)$$

When finding the equilibrium human model states for a given mean EIR ε_0, the equilibrium is calculated for EIRs $\zeta_1\varepsilon_0,\ldots,\zeta_n\varepsilon_0$ and then the weighted average of each model state is found using the weights $w_1,\ldots,w_n$. So for simplicity the remainder of the description of how to find the equilibrium human states does not explicitly mention this heterogeneity in exposure, i.e. it is conditional on an EIR $\varepsilon = \zeta_j\varepsilon_0$ for some $j = 1,\ldots,n$.

Ageing

Ageing is modelled using age groups with exponential transitions between each group. There is a constant death rate η, balanced by the birth rate. Suppose that there are N age groups $[a_{i,}a_{i+1})$ for $i = 1,\ldots,N$ with $a_1 = 0$ and $a_{N+1} = \infty$, and that each contains a proportion of the population π_i. The width can vary, so that for example there are finer groups at young ages, to more accurately describe the peak in incidence of disease. Then the rate of exiting age group i to the next age group is $r_i = 1/(a_{i+1} - a_i),\ i = 1,\ldots,N-1;\ r_N = 0$. Defining $r_0 = \eta$ and $\pi_0 = 1$,

$$\frac{d\pi_i}{dt} = r_{i-1}\pi_{i-1} - (r_i + \eta)\pi_i$$

This has equilibrium solution (the exact equilibrium for this model with compartmental age groups):

$$\pi_i = \frac{r_{i-1}\pi_{i-1}}{(r_i + \eta)}$$

If the mean EIR for adults is ε, then the EIR and force of infection in age group i are

$$\varepsilon_i = \varepsilon\psi_i,\ \Lambda_i = \varepsilon_i b_i$$

where ψ_i is the relative biting rate by age and b_i is the probability of infection.

Immunity functions

Partial immunity which reduces the probability of infection, of clinical malaria and of parasites being detected is modelled using an empirical function for each kind of immunity. These functions increase with exposure and decay in the absence of exposure. For each immunity function I, the partial differential equation over time and age has the form

$$\frac{\partial I}{\partial t} + \frac{\partial I}{\partial a} = F - I/d$$

F is a quantity that depends on exposure and d is a parameter, with both F and d being different for each type of immunity. Full details are given in [1].

When ageing is modelled using discrete groups, I_i is the mean value of I in age group i. Ignoring F and d at first, for a small time step Δ, defining I_0 to be the value of I at birth, and assuming that the age distribution is in equilibrium:

$$\begin{aligned} I_i(t+\Delta) &= \frac{\left(1-\Delta(r_i+\eta)\right)\pi_i I_i(t) + \Delta r_{i-1}\pi_{i-1}I_{i-1}(t)}{\left(1-\Delta(r_i+\eta)\right)\pi_i + \Delta r_{i-1}\pi_{i-1}} \\ &= I_i(t) + \Delta\left(r_{i-1}\pi_{i-1}/\pi_i I_{i-1}(t) - (r_i+\eta)I_i(t)\right) \\ &= I_i(t) + \Delta(r_i+\eta)\left(I_{i-1}(t) - I_i(t)\right) \end{aligned}$$

Hence the dynamics due to ageing are:

$$\frac{dI_i}{dt} = (r_i+\eta)\left(I_{i-1} - I_i\right)$$

Adding the increase with exposure and subsequent waning, we have

$$\frac{dI_i}{dt} = F_i - I_i / d + (r_i+\eta)\left(I_{i-1} - I_i\right)$$

and in equilibrium

$$I_i = \frac{F_i + (r_i+\eta)I_{i-1}}{1/d + (r_i+\eta)}, i = 1,\ldots,N$$

For anti-infection immunity I_B , F depends on the EIR:

$$F_{Bi} = \frac{\varepsilon_i}{\varepsilon_i u_B + 1}$$

While for acquired clinical immunity I_{CA} and anti-parasite immunity I_D , F depends on the force of infection:

$$F_{Ci} = \frac{\Lambda_i}{\Lambda_i u_C + 1}, F_{Di} = \frac{\Lambda_i}{\Lambda_i u_D + 1}$$

u_B, u_C and u_D are parameters determining the maximum rate at which immunity can be acquired.

For anti-infection immunity, acquired clinical immunity and anti-parasite immunity the value at birth is 0. Maternal clinical immunity decays from birth, i.e. there is no increase with exposure, $F = 0$.

So to find the equilibrium human model states conditional on a given EIR, the procedure is to first find the equilibrium immunity to infection by age, from which the probability of infection b_i and hence the force of infection by age can be found. Then the probability of clinical malaria by age

group can be found, denoted by ϕ_i, as can the probability of detection of asymptomatic infections, q_i. Each of these three probabilities (b_i, ϕ_i and q_i) is a transformation of the corresponding immunity function as detailed in [1].

Equilibrium infection states

There are six human infection states: susceptible (S), treated clinical disease (T), untreated clinical disease (D), asymptomatic infection which may be detected by microscopy (A), sub-patent infection (U) and protected by a period of prophylaxis from prior treatment (P).

The equations for the infection states in age group i are:

$$\frac{dS_i}{dt} = -\beta_{Si}S_i + r_{i-1}S_{i-1} + r_P P_i + r_U U_i$$
$$\frac{dT_i}{dt} = -\beta_{Ti}T_i + r_{i-1}T_{i-1} + f_T\phi_i\Lambda_i(S_i + A_i + U_i)$$
$$\frac{dD_i}{dt} = -\beta_{Di}D_i + r_{i-1}D_{i-1} + (1-f_T)\phi_i\Lambda_i(S_i + A_i + U_i)$$
$$\frac{dA_i}{dt} = -\beta_{Ai}A_i + r_{i-1}A_{i-1} + (1-\phi_i)\Lambda_i(S_i + U_i) + r_D D_i$$
$$\frac{dU_i}{dt} = -\beta_{Ui}U_i + r_{i-1}U_{i-1} + r_A A_i$$
$$\frac{dP_i}{dt} = -\beta_{P_i}P_i + r_{i-1}P_{i-1} + r_T T_i$$

where

$$\beta_{Si} = \Lambda_i + r_i + \eta$$
$$\beta_{Ti} = r_T + r_i + \eta$$
$$\beta_{Di} = r_D + r_i + \eta$$
$$\beta_{Ai} = \phi_i\Lambda_i + r_A + r_i + \eta$$
$$\beta_{Ui} = \Lambda_i + r_U + r_i + \eta$$
$$\beta_{Pi} = r_P + r_i + \eta$$

and defining $S_{-1} = 1$ and $T_{-1} = D_{-1} = A_{-1} = U_{-1} = P_{-1} = 0$. f_T is the proportion of symptomatic infections that are effectively treated. r_D, r_T, r_A, r_U and r_P are the recovery rates from the respective infection states.

Put $Y_i = S_i + A_i + U_i$. Then

$$\frac{dY_i}{dt} = -\beta_{Yi} Y_i + r_{i-1} Y_{i-1} + r_P P_i + r_D D_i$$
$$\frac{dT_i}{dt} = -\beta_{Ti} T_i + r_{i-1} T_{i-1} + f_T \phi_i \Lambda_i Y_i$$
$$\frac{dD_i}{dt} = -\beta_{Di} D_i + r_{i-1} D_{i-1} + (1 - f_T) \phi_i \Lambda_i Y_i$$
$$\frac{dP_i}{dt} = -\beta_{Pi} P_i + r_{i-1} P_{i-1} + r_T T_i$$

where

$$\beta_{Yi} = \Lambda_i \phi_i + r_i + \eta$$

In equilibrium

$$T_i = \frac{f_T \phi_i \Lambda_i Y_i + r_{i-1} T_{i-1}}{\beta_{Ti}}$$
$$D_i = \frac{(1 - f_T) \phi_i \Lambda_i Y_i + r_{i-1} D_{i-1}}{\beta_{Di}}$$
$$P_i = \frac{r_T T_i + r_{i-1} P_{i-1}}{\beta_{Pi}}$$

Or

$$T_i = a_{Ti} Y_i + b_{Ti}$$
$$D_i = a_{Di} Y_i + b_{Di}$$
$$P_i = a_{P_i} Y_i + b_{Pi}$$

where

$$a_{Ti} = \frac{f_T \phi_i \Lambda_i}{\beta_{Ti}}, \ b_{Ti} = \frac{r_{i-1} T_{i-1}}{\beta_{Ti}}$$
$$a_{Di} = \frac{(1 - f_T) \phi_i \Lambda_i}{\beta_{Di}}, \ b_{Di} = \frac{r_{i-1} D_{i-1}}{\beta_{Di}}$$
$$a_{Pi} = \frac{r_T a_{Ti}}{\beta_{Pi}}, \ b_{Pi} = \frac{r_T b_{Ti} + r_{i-1} P_{i-1}}{\beta_{Pi}}$$

The total proportion in age group i is

$$\pi_i = Y_i + T_i + D_i + P_i$$

Rearranging this gives

$$Y_i = \frac{\pi_i - (b_{Ti} + b_{Di} + b_{Pi})}{1 + a_{Ti} + a_{Di} + a_{Pi}}$$

which can be used to find T_i, D_i, and P_i. Then the remaining states can be found:

$$A_i = \frac{r_{i-1}A_{i-1} + (1-\phi_i)\Lambda_i Y_i + r_D D_i}{\beta_{Ai} + (1-\phi_i)\Lambda_i}$$

$$U_i = \frac{r_A A_i + r_{i-1}U_{i-1}}{\beta_{Ui}}$$

$$S_i = Y_i - A_i - U_i$$

Force of infection on mosquitoes

Once the equilibrium infection states by age and heterogeneity level have been found, the force of infection on mosquitoes Λ_M can be calculated. The model states now include the subscript j for the heterogeneity classes and denote the proportion within each heterogeneity class in each model state and age group, so that summed over ages and model states they add up to 1 within each heterogeneity class.

$$\Lambda_M = \sum_{j=1}^{n}\sum_{i=1}^{N}\left(D_{ij}c_D + T_{ij}c_T + A_{ij}c_{Aij} + U_{ij}c_U\right)\alpha_{ij}w_j$$

$\alpha_{ij} = \alpha_0 \frac{\psi_i}{\omega}\zeta_j$ is the rate at which people are bitten in age group i, heterogeneity level j, with α_0 the overall mean biting rate. Each c is the probability of infecting a susceptible mosquito when in the corresponding infection state. c_{Aij} depends on immunity, as a transformation of q_{ij}, the probability that an infection will be detected , with γ_I an additional parameter:

$$c_{Aij} = c_U + (c_D - c_U)q_{ij}^{\gamma_I}$$

Onward infectiousness with no immunity

The formula in equation (4) of the main text for R_0 for the Griffin model does not account for the fact that people age during an infection, and so the rate at which they are bitten by mosquitoes changes. Let $c(t)$ be the expected infectiousness at time t after blood-stage infection appears. Accounting for ageing during an infection gives:

$$\begin{aligned}
R_0 &= m\gamma b \int_0^\infty \int_0^\infty \zeta \frac{\psi(a)}{\omega} \alpha_0 \, h(\zeta) g(a) \int_0^\infty \zeta \frac{\psi(a+d_E+t+t_l)}{\omega} \alpha_0 \, e^{-(d_E+t_l)\eta} c(t)\, dt\, d\zeta\, da \\
&= m\gamma b \delta_h {\alpha_0}^2 B \\
B &= \frac{1}{\omega^2} \int_0^\infty \psi(a) g(a) \int_0^\infty \psi(a+d_E+t+t_l) e^{-(d_E+t_l)\eta} c(t)\, dt\, da \\
\delta_h &= \int_0^\infty \zeta^2 h(\zeta)\, d\zeta
\end{aligned}$$

d_E is the duration of the latent infection stage and t_l is the time lag from asexual parasite states to the onwards infectiousness resulting from those states. Gametocytes are not explicitly modelled, and t_l is taken as a fixed duration rather than a distribution.

In the formula in the main text, B is approximated by

$$\begin{aligned}
B &\simeq \frac{1}{\omega^2} \int_0^\infty \psi(a) g(a) \int_0^\infty \psi(a) c(t)\, dt\, da = \delta_a C \\
\delta_a &= \int_0^\infty \left(\psi(a)/\omega\right)^2 g(a)\, da, \; C = \int_0^\infty c(t)\, dt
\end{aligned}$$

This is accurate to within around 1% of its correct value with the fitted model parameters.

To evaluate B exactly, as was done for the results in the main text, $c(t)$ can be expressed as the sum over the different infection states of the probability of being in that state at time t after blood-stage infection occurs multiplied by the infectiousness if in that state:

$$\begin{aligned}
c(t) = {} & \phi f_T p_{T,T}(t) c_T + \\
& \phi(1-f_T)\left(p_{D,D}(t) c_D + p_{D,A}(t) c_A + p_{D,U}(t) c_U \right) + \\
& (1-\phi)\left(p_{A,A}(t) c_A + p_{A,U}(t) c_U \right)
\end{aligned}$$

$p_{D,A}(t)$ is the probability of being in state A at time t after blood-stage infection occurs, if a person initially entered state D, and similarly for the other possible combinations of states. With no immunity, $c_A = c_D$.

These probabilities are:

$$p_{T,T}(t) = e^{-(r_T+\eta)t}$$
$$p_{D,D}(t) = e^{-(r_D+\eta)t}$$
$$p_{D,A}(t) = \frac{r_D}{r_A - r_D}\left(e^{-(r_D+\eta)t} - e^{-(r_A+\eta)t}\right)$$
$$p_{D,U}(t) = \frac{r_A r_D}{r_A - r_D}\left(\frac{\left(e^{-(r_D+\eta)t} - e^{-(r_U+\eta)t}\right)}{r_U - r_D} - \frac{\left(e^{-(r_A+\eta)t} - e^{-(r_U+\eta)t}\right)}{r_U - r_A}\right)$$
$$p_{A,A}(t) = e^{-(r_A+\eta)t}$$
$$p_{A,U}(t) = \frac{r_A}{r_U - r_A}\left(e^{-(r_A+\eta)t} - e^{-(r_U+\eta)t}\right)$$

$c(t)$ can be written in the following form:

$$c(t) = \sum_k \theta_k e^{-\rho_k t}$$

Hence B can be found by integrating each exponential term twice:

$$\int_0^\infty \psi(a + d_E + t + t_l) e^{-(d_E+t_l)\eta} c(t)\, dt = \sum_k e^{-(d_E+t_l)\eta}\theta_k \int_0^\infty \left(1 - \rho e^{-(a+d_E+t+t_l)/a_0}\right) e^{-\rho_k t}\, dt$$
$$= \sum_k e^{-(d_E+t_l)\eta}\theta_k \left(\frac{1}{\rho_k} - \frac{\rho e^{-(a+d_E+t_l)/a_0}}{\rho_k + 1/a_0}\right)$$

$$B = \frac{1}{\omega^2}\int_0^\infty \left(1 - \rho e^{-a/a_0}\right)\eta e^{-\eta a}\sum_k e^{-(d_E+t_l)\eta}\theta_k \left(\frac{1}{\rho_k} - \frac{\rho e^{-(a+d_E+t_l)/a_0}}{\rho_k + 1/a_0}\right) da$$
$$= \frac{e^{-(d_E+t_l)\eta}}{\omega^2}\sum_k \theta_k \int_0^\infty \left(1 - \rho e^{-a/a_0}\right)\eta e^{-\eta a}\left(\frac{1}{\rho_k} - \frac{\rho e^{-(a+d_E+t_l)/a_0}}{\rho_k + 1/a_0}\right) da$$
$$= \frac{e^{-(d_E+t_l)\eta}}{\omega^2}\sum_k \theta_k \left(\frac{1}{\rho_k} - \frac{\eta\rho}{\rho_k(\eta + 1/a_0)} - \frac{\eta\rho e^{-(d_E+t_l)/a_0}}{(\rho_k + 1/a_0)(\eta + 1/a_0)} + \frac{\eta\rho^2 e^{-(d_E+t_l)/a_0}}{(\rho_k + 1/a_0)(\eta + 2/a_0)}\right)$$

References

1. Griffin J.T., Ferguson N.M., Ghani A.C. 2014 Estimates of the changing age-burden of Plasmodium falciparum malaria disease in sub-Saharan Africa. *NATURE COMMUNICATIONS* **5**. (doi:10.1038/ncomms4136).

2. Abramowitz M., Stegun I.A. 1972 Handbook of Mathematical Functions with Formulas, Graphs, and Mathematical Tables. National Bureau of Standards Applied Mathematics Series 55. Tenth Printing.
